# The Tuberculosis Congress

**Published:** 1901-07-13

**Authors:** 


### The Hospital

A Journal of

### The Medical Sciences and Hospital Administration.

Vol. XXX.—No. 772.] Edited by Sir Henry Burdett, K.C.B., and Solomon C. Smith, M.D., M.R.C.P. [July 13, 1901.

### The Tuberculosis Congress.

The Congress which will shortly meet to discuss the great problems which arise out of the prevalence of tuberculous diseases will be by no means exclusively a medical affair. Medicine has indeed said its say. It has been shown that tuberculosis is due to the development of a parasitic organism, and that it is spread by whatever facilitates the transfer of the parasite from man to man and by whatever renders man susceptible to its attack. It has been shown also by what means the infection is chiefly spread, and what are the conditions by which a predisposition to its attacks is generally established, and so far as this, the medical, side of the question is concerned, it may be doubted whether the Congress will carry matters much further. The real problem which the Congress may usefully attack is the sociological one, including, on the one hand, the question how to remove those conditions which are known to favour the occurrence of tuberculosis, and on the other how to prevent the dissemination of infection. Those who take long views can have no doubt that, for the final eradication of tuberculosis, the only really effective measures must be those which go to eradicate the conditions which favour its development—the crowded homes, the want of light and air, the dirt, the dust, the drink, the vice, the innutrition, and the whole category of insanitary conditions which at present so largely prevail in the dwellings and in too many of the workplaces of the poor. To the individual tuberculosis is the result of an infection, but to the community the prevalence of tuberculosis means the prevalence of insanitary modes of life, taking the word insanitary in its widest sense, and to those who look to the future the best means for preventing consumption appear to be those which aim at improving the whole standard of life. Most of us, however, take short views and ask for quick if not immediate results, and those of us who ask that something shall be done to remedy the condition of affairs which actually exists find ourselves confronted with "infection" as the problem which has to be attacked, and it is because interference with the dissemination of infection involves a large interference with man in his daily life, not merely with man as he lies on a sick bed, but with man as a breadwinner, that the problem of tuberculosis is so fitting a one for a Congress which is largely sociological. For many liberties are at stake.

As with every other zymotic disease infection of tuberculosis may arise from various sources, but it is hardly to be doubted that its common source is infected man. It is worth while insisting somewhat upon this point in view of the coming Congress, because the crusade against consumption is at the present time being regarded from so many sides, and, if we may venture to say so, is being used by so many interests that the essentials are somewhat apt to be hidden by side issues. Those who are interested in the dwellings of the poor use the prevalence of tuberculosis as a lever for their cause; those who wish to reform the slaughter-houses trust to tuberculosis to point the moral of their tale; those who aim at bringing the milk trade under more strict control find in the spread of infantile tuberculosis a tool ready to their hands; while even those who object to modern methods of sewage disposal seek in the persistence of "acid-fast" bacteria in sewage effluents a weapon to turn against the sanitary engineer. And so on.

But although these people are all right, each in his own way, no one can carefully regard the evidence without seeing that, as things stand at the present time, the sufferer from chronic phthisis, the man or woman who goes on year after year, sometimes at work, sometimes laid up, but always a possible source of infection to others, is the real difficulty which stands in the way of our crusade against the disease. And this is the real question which a great Congress may profitably discuss from social and economic points of view. We put on one side acute miliary tuberculosis and acute pneumonic phthisis. These are diseases which quickly lay the sufferer low and keep him under medical supervision and control. They could without any material wrench to public feeling or general custom be fully dealt with by a slight extension of the existing sanitary machinery of the country. But chronic phthisis is another affair. A gradual decline from health, broken, no doubt, by intercurrent attacks of illness, but during the larger portion of its course, that is, through several years, leaving the patient able and willing to work, a useful citizen, bearing his part in the social life of his town or village, a breadwinner, the father of a family, often, horrible to say, the father of an increasing family, and yet all the time a possible source of infection to those among whom he works and lives.

To deal with those actually sick is a mere question of money derived either from charity or rates. Now that we know of a way by which cases of incipient phthisis may be restored to health, it almost goes without saying, in this humanitarian age, that this way should be opened by the establishment of sanatoria for the treatment of those who are attacked; while, now that we know that patients in the later and more helpless stages of consumption are a source of great danger to those with whom they live, so long as they live amid the ordinary surroundings of poverty, it again goes without saying that refuges for such people should be opened, hospitals in which these helpless ones, the waste and wreckage of an imperfect civilisation, may pass their last days in comfort and without endangering the lives of others. To provide these means of cure and of relief is no doubt a big business. After all, however, it is but a question of money. But what to do with the working man, the clerk, the journalist, the editor, the society lady, the seamstress, the mother of an increasing family, all self-supporting or independent and each filling his or her own place in life—each a free and independent citizen, and yet each a possible source of infection—there is the nut for the Congress to crack.

